# Diagnostic Accuracy of Golgi Protein 73 (GP73) for Liver Fibrosis Staging in Metabolic Dysfunction-Associated Steatotic Liver Disease: A Scoping Review and Cohort Study

**DOI:** 10.3390/diagnostics15050544

**Published:** 2025-02-24

**Authors:** Valentina Pecoraro, Fabio Nascimbeni, Michela Cuccorese, Filippo Gabrielli, Tommaso Fasano, Tommaso Trenti

**Affiliations:** 1 Complex Structure of Laboratory Medicine, Department of Laboratory Medicine and Pathological Anatomy, AUSL Modena, 41121 Modena, Italy; 2 Metabolic Medicine Unit, AOU Modena, 41124 Modena, Italy; 3 Bianalisi Healthcare Group, 20841 Carate Brianza, Italy

**Keywords:** Golgi protein 73, metabolic dysfunction-associated steatotic liver disease, accuracy

## Abstract

**Background/Objectives**: Golgi protein 73 (GP73) is a transmembrane protein expressed by epithelial cells of the bile duct in the normal liver. High serum levels of GP73 have been detected in patients with acute or chronic liver diseases, MASLD, and its measurement has been suggested as a potential biomarker for liver fibrosis staging. We evaluated the utility of GP73 in the diagnosis of MASLD, MASH, and for liver fibrosis staging. **Methods**: We performed a literature scoping review to map the current evidence about the accuracy of GP73 in patients with MASLD. We searched in Medline and EMBASE for English studies reporting an AUC value of GP73 in diagnosing MASLD and MASH and evaluating GP73 for fibrosis staging. A narrative synthesis of the evidence was conducted. Moreover, we performed an observational study including 84 patients with MASLD, of which 60 were biopsy-confirmed MASH, and different liver fibrosis stages, and 15 healthy controls. Serum GP73 levels were determined using a chemiluminescent assay and reported as mean and standard deviation (SD). Sensitivity (SE), specificity (SP), the area under the receiver operating characteristic (AUROC) curve, and the optimal cut-off value were calculated. Data were considered statistically significant when *p* < 0.05. **Results:** Available studies evaluating GP73 in MASLD reported the ability to discriminate MASH from simple steatosis and distinguish patients at different fibrotic stages, but the evidence is still scarce. Our experimental study showed that the serum levels of GP73 were 30 ± 12 ng/mL in MASLD and 32 ± 12 ng/mL in MASH patients and were statistically higher than those of the control group (19 ± 30 ng/mL), increasing from liver fibrosis stage F0 to F4. GP73 levels were significantly higher in patients with significant and advanced fibrosis than controls and no significant fibrosis (*p* > 0.05). ROC analysis demonstrated that serum GP73 had a good diagnostic potential for MASLD (AUROC 0.85; SE 90%; SP 73%), MASH (AUROC 0.75; SE 82%; SP64%), and significant fibrosis (AUROC 0.7; SE 56%; SP 79%) and was better than other biomarkers for chronic liver diseases. **Conclusions**: Serum GP73 could support clinicians in the evaluation of patients with MASH and significant fibrosis.

## 1. Introduction

Non-alcoholic fatty liver disease (NAFLD) is the most common chronic liver disease worldwide, with a prevalence of about 30% in adults [1]. NAFLD is defined by the presence of steatosis in more than 5% of hepatocytes in association with metabolic risk factors such as obesity and type 2 diabetes and in the absence of excessive alcohol consumption and other causes of chronic liver disease [2]. NAFLD is a heterogeneous disorder including a wide spectrum of conditions, from simple steatosis with or without inflammation (non-alcoholic fatty liver—NAFL) to non-alcoholic steatohepatitis (NASH) with different stages of fibrosis until cirrhosis and hepatocellular carcinoma (HCC). The pathogenic pathways of NAFLD are influenced by multiple metabolic, genetic, and microbiome-related factors that are not completely understood [2]. In 2023, the terms NAFLD and NASH were changed to metabolic dysfunction-associated steatotic liver disease (MASLD) and metabolic dysfunction-associated steatohepatitis (MASH), respectively, to highlight the weight of metabolic and cardiovascular factors in the pathogenesis of these liver disorders [3]. Patients with MASLD/MASH have a high risk of developing cirrhosis and HCC, and the severity of liver fibrosis is the main histologic predictor of adverse liver-related outcomes. In particular, severe fibrosis and cirrhosis were associated with an increased risk of liver-related complications and mortality for all causes [4]. In this context, the evaluation of liver fibrosis stage is needed to assess disease severity and provide prognostic information. Liver biopsy is the gold standard for fibrosis assessment, but it has some limitations due to its invasive nature [5]. Several non-invasive tests (NITs), including direct and indirect serum biomarkers, have been evaluated to assess the severity of liver fibrosis [6].

Golgi protein 73 (GP73) is a type II Golgi transmembrane protein with a molecular weight of 73 kDa. GP73 is mainly expressed by epithelial cells of the bile duct in the normal liver and also in various human tissues, such as the prostate, stomach, colon, trachea, adrenal gland, and pituitary gland, with minimal to no expression in the hepatocytes of healthy livers. Instead, in liver diseases, regardless of the underlying etiology, the GP73 expression in hepatocytes is markedly increased. High serum levels of GP73 have been detected in acute and chronic liver diseases in both viral (HBV, HCV) and non-viral etiologies (metabolic, autoimmune), and it is markedly upregulated in HCC [7]. In the course of chronic liver disease, serum GP73 levels gradually increase with the progression of liver fibrosis and the development of cirrhosis and HCC, but this elevation is reversible after disease remission [8].

Several studies showed that GP73 promotes chronic inflammation by upregulating the secretion of pro-inflammatory cytokines, especially Il-6, which in turn promotes the proliferation of hepatic stellate cells (HSCs), whose activation is a pivotal event for liver fibrosis development. Although the relationship between GP73 and inflammatory pathways is highly complex, and the mechanisms underlying the fibrosis process have not yet been clarified, several studies suggested the involvement of GP73 in the disease progression of fibrosis and cirrhosis [8]. Increased serum GP73 levels seem to be triggered under inflammatory conditions in the case of HCV and HBV infection, autoimmune disease, and cancer. GP73 is highly expressed in HCC patients, where it leads to pro-inflammatory cytokine production, differentiation, and growth of T lymphocytes and macrophage cells, facilitating tumor progression [9]. Moreover, some authors reported that GP73 was positively correlated with other liver-specific biomarkers such as hyaluronic acid (HA) and collagen type IV (CIV), and its accuracy was comparable to AST/PLT ratio (APRI) assay, indicating their potential roles in liver fibrosis [10].

These findings suggest that GP73 could be a promising biomarker for the diagnosis of liver diseases and for distinguishing patients at different fibrotic stages. To date, the diagnostic value of GP73 in patients with MASLD has been evaluated in few studies. We performed a literature scoping review to map the current evidence about the accuracy of GP73 in patients with MASLD. Furthermore, we performed an experimental study to evaluate: a) the serum levels of GP73 in a cohort of biopsy-confirmed MASLD patients, and b) the diagnostic accuracy of GP73 to discriminate patients with MASH and significant or advanced fibrosis.

## 2. Materials and Methods

### 2.1. Literature Scoping Review

We searched PubMed and EMBASE from inception to November 2024 to identify studies evaluating the clinical use of GP73 in patients with MASLD (Supplementary Table S1). There were no restrictions regarding the date of publication. We used Medical Subject Heading (MeSH) terms and free text words (including synonyms and closely related words) related to GP73, NAFLD, and liver fibrosis. To avoid omitting potentially eligible studies, we also manually searched the reference lists of the included studies, relevant reviews, and relevant meta-analyses. The reporting adheres to the Preferred Reporting Items for Systematic Reviews and Meta-Analyses Scoping Review (PRISMA-ScR) guidelines [11]. The screening of the studies was divided into two stages: first, title and abstract screening; then, full-text screening. Two researchers then independently reviewed the titles and abstracts based on the Population, Intervention, Comparison, Outcomes, and Study Design (PICOS) criteria to determine the eligibility of each article. Disagreements between the researchers were resolved through discussions. Then, two reviewers screened all the full texts and selected studies, considering the inclusion criteria, and generated a list of excluded studies and the reasons for exclusion.

We included studies that meet the following inclusion criteria: (a) randomized controlled trials (RCTs), non-randomized controlled studies, observational and cross-sectional studies, prospective and retrospective studies; (b) reported the serum concentration of GP73; (c) included adult patients (over 18 years old) with MASLD or children; (d) reported diagnostic accuracy data (AUC, sensitivity, specificity); (e) liver biopsy was used as the reference standard; (f) written in English. We excluded studies unrelated to the topic, basic or animal research reviews, meta-analyses, conference abstracts, comments, editorials, and letters.

One reviewer used a standardized form to extract data from the included studies. The following data were extracted: author, publication year, study design, country, sample size, mean age of participants, stage of fibrosis, GP73 concentration, reference standard, sensitivity (SE), specificity (SP), and area under the curve (AUC) value for GP73. To ensure the accuracy of the extracted data, a second reviewer checked the extracted data.

Two researchers independently assessed the methodological quality of the included studies. Diagnostic studies were evaluated with the Quality Assessment of Diagnostic Accuracy Studies (QUADAS-2) tool [12]. Four domains were considered (patient selection, index test, reference standard, and flow and timing), each rated in terms of their risk of bias and applicability to the research question. Risk of bias and applicability were judged as “low”, “high”, or “unclear”. Any disagreements were resolved through discussion.

For each included study, we described the main characteristics in detail and tabulated the quantitative data. A narrative synthesis of the evidence was provided.

### 2.2. Experimental Study

The study was conducted in accordance with the International Coordinating Council for Clinical Trials and the Helsinki Declaration. Reporting was compliant with STARD guidelines [13].

We collected consecutive serum samples from patients admitted to the Internal and Metabolic Medicine unit of Modena who were submitted for liver biopsy for the suspicion of MASH with advanced fibrosis. We enrolled adult patients (over 18 years old) of both genders with any grade of liver fibrosis. The diagnosis of MASLD was based on ultrasonographic/biopsy-proven fatty liver in the absence of excessive alcohol consumption (defined as daily alcohol intake > 20 g) and other competing etiologies of liver disease [14]. Criteria for exclusion from the study were as follows: (a) diagnosis of alcoholic liver disease, viral (HCV or HBV-related) chronic liver disease or other liver diseases (autoimmune); (b) pregnancy; (c) the presence of decompensated cirrhosis; (d) either primary or metastatic liver cancer or other tumors.

Additionally, we collected a serum sample from 15 healthy volunteers without acute or chronic diseases, hyperlipidemia, diabetes, or hypertension as negative controls. All serum samples were collected, centrifuged, and stored at −20 °C for serological analysis. Biopsy specimens of patients with MASLD were scored according to the Brunt and Kleiner criteria [15,16]. Fibrosis was staged (F0 = none; F1 = perisinusoidal/pericellular or portal/periportal; F2 = perisinusoidal/pericellular plus portal/periportal; F3 = bridging; F4 = cirrhosis). Liver fibrosis was considered as follows: (a) no significant presence of fibrosis stages F0 and F1 only; (b) minor presence of fibrosis stages F0, F1, and F2; (c) significant presence of fibrosis stages ≥ F2; and (d) advanced presence of fibrosis stages ≥ F3 [17].

We collected demographic (age, gender) and clinical (including liver biopsy data: MASH diagnosis, NAS score, and stage of liver fibrosis) data. Furthermore, details about the measures of non-invasive tests (NITs) (AST, ALT, GGT, platelet count, CIV, HA, and PIIIPNP) were collected retrospectively. Moreover, AST/ALT ratio and AST to platelet ratio index (APRI) values were calculated.

Serum GP73 was measured for all serum samples on a Maglumi X8 (Snibe Co., Ltd., Shenzhen, Cina) platform using chemiluminescent microparticle immunoassay (CLIA) technology. The procedures and the interpretation of the results were performed according to the manufacturer’s instructions. Patient samples were incubated with magnetic microspheres coated with monoclonal anti-GP73 antibodies to form an antigen–antibody complex. Under a magnetic field, magnetic particles are absorbed into the inner wall of reaction tubes, and unbound material is washed away from the solid phase. Then, ABEI-labelled anti-GP73 antibodies are added and incubated to form a sandwich compound. The subsequent addition of starters determines the start of the chemiluminescent reaction. The light signal is measured by a photomultiplier in the form of relative light units (RLUs) proportional to the concentration of GP73 present in the sample. The concentration of GP73 is automatically calculated according to the RLUs and a built-in calibration curve, and a concentration ≥ 45 ng/mL is indicated by manufacturers as a positive result.

Results were expressed as mean and standard deviation (SD). Relationships between continuous variables were analyzed using Spearman’s correlation. A contingency table was created to calculate sensitivity (SE), specificity (SP), and likelihood ratio positive (LR+) and negative (LR−) for GP73. The area under the receiver operating characteristic (AUROC) curve and the optimal cut-off value were also calculated using Youden’s index. Data were considered statistically significant when *p* < 0.05. Analyses were performed using MedCalc v.23.0.9 (MedCalc Software Ltd., Ostend, Belgium) software.

## 3. Results

### 3.1. Literature Scoping Review

**Figure 1:**
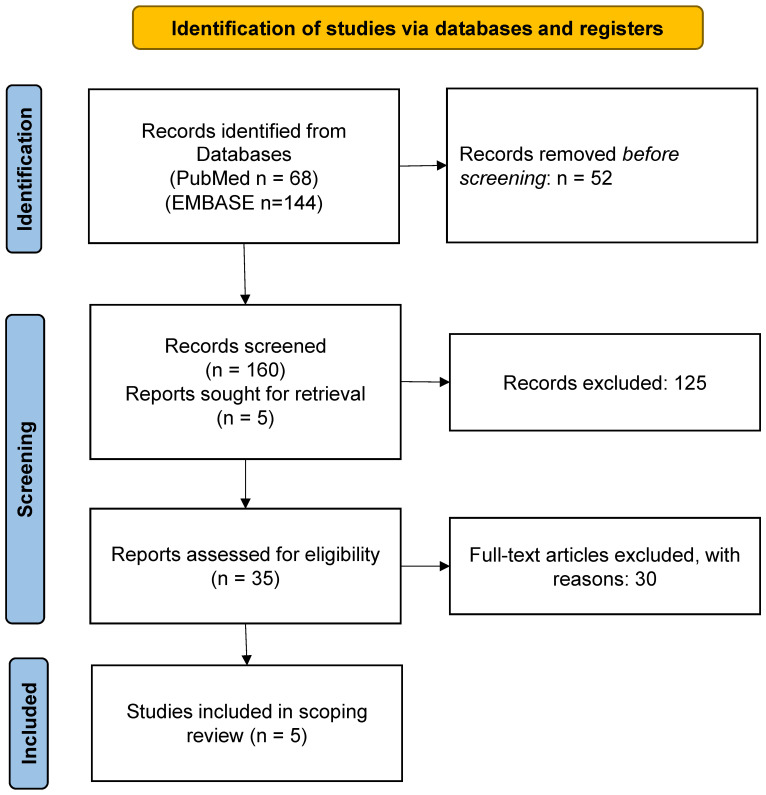
PRISMA flow diagram of the literature review.

The literature search identified 212 references, of which 35 were considered eligible, and the full texts were scrutinized. Of these, 30 were excluded because they did not meet the inclusion criteria [7,8,18,19,20,21,22,23,24,25,26,27,28,29,30,31,32,33,34,35,36,37,38,39,40,41,42,43,44,45] (Supplementary Table S2). Finally, full-text screening resulted in the inclusion of five studies [10,46,47,48,49] (Figure 1).

The main characteristics of the included studies are reported in Table 1. All included studies had been performed in China, four were prospective, and one was a retrospective cohort study. Overall, the studies included 780 patients with MASLD, of which 486 (62.3%) were classified as MASH. Four studies included adults, and one study [10] included pediatric participants.

**Figure 2:**
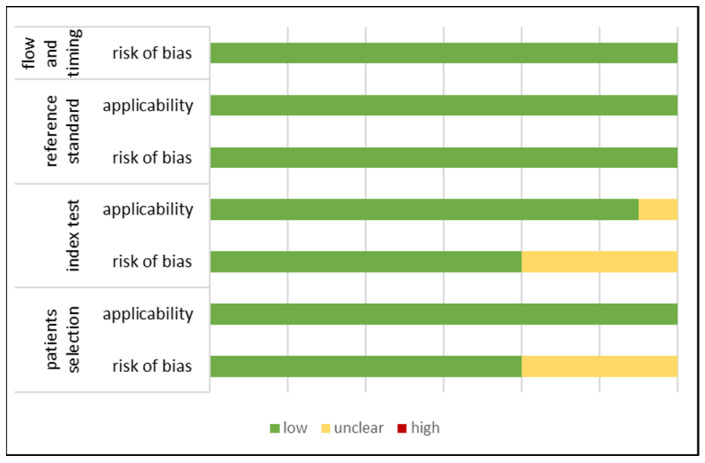
Summary of risk of bias assessment with the QUADAS-2 tool. The x-axis represents the percentage of studies graded to a specific risk of bias: low, moderate, or high risk of bias. The y-axis represents the four domains that were graded: patient selection, index test, reference standard, flow and timing.

We assessed the risk of bias for all studies with the QUADAS-2 tool. Three studies enrolled consecutive patients, one study was retrospective, and in another study the enrolment was unclear. In four studies, the authors were blinded to the results of the index test and reference standard, and all studies reported a prespecified threshold value. In all included studies, the time interval between the index test and reference standard was appropriate, and all patients received the same reference standard (Figure 2, Supplementary Table S3).

Li et al. [46] evaluated the utility of GP73 in the diagnosis of MASH and hepatic fibrosis staging. They reported that the serum GP73 concentrations of the NAFL and MASH groups were significantly higher than those of controls (*p* < 0.05) and were significantly different (*p* < 0.05) in consideration of the different severities of hepatic fibrosis. Serum GP73 positively correlated with liver expression. Moreover, there was a strong positive correlation of the combination of ALT, GGT, and GP73 with MASH. For the diagnosis of MASH, the AUROC curves for serum GP73 in participants with MASLD was 0.83, and for the identification of liver fibrosis stage F ≥ 2, the sensitivity and specificity were optimal (Table 1). The authors concluded that serum GP73 may represent a useful serological biomarker for the diagnosis and monitoring of MASLD patients.

Liu et al. [10] evaluated GP73 in the liver and serum of children of age < 18 years. The authors showed that serum GP73 levels decreased with age in both subgroups (patients and controls) and that the serum concentration was higher in patients below or older than 3 years than in controls. Serum levels of GP73 were gradually elevated with the progression of liver fibrosis and were significantly higher in patients with significant fibrosis compared to patients with no/minor fibrosis in the subgroup aged less than 3 years. No significant difference between no/minor fibrosis and significant fibrosis in the subgroup aged 3 years or older was observed. Likewise, serum GP73 levels were significantly higher in patients with significant inflammation than those of patients with no/minor inflammation in a subgroup aged less than 3 years and a subgroup aged 3 years or older. The AUROC of serum GP73 for diagnosing significant fibrosis was suboptimal in patients aged 3 years (0.6) and optimal in patients aged below 3 years (0.76) (Table 1).

Wang et al. [47] evaluated the diagnostic potential of GP73 for hepatic necroinflammation in MASH patients. The authors reported that serum levels of GP73 were significantly different between patients with different grades of hepatic necroinflammation and were higher in patients with severe activity (*p* < 0.05) (Table 1). Moreover, a gradual increase of GP73 protein expression in situ was also observed in liver tissue, in parallel with the increasing severity of necroinflammatory activity. The serum GP73 correlated well with the intensity of protein expression in liver tissue. In addition, with the AUROC as 0.89 and 0.74, the serum GP73 exhibited excellent performance in identifying severe (G = 3) and moderate (G ≥ 2) inflammatory activity in MASH patients. The authors concluded that GP73 is a possible alternative serum marker reflecting the severity of hepatic necroinflammation in MASH patients.

Yao et al. [48] examined serum GP73 in diagnosing chronic liver disease both in cirrhotic and pre-cirrhotic subjects. In the study, the authors reported that serum levels of GP73 were significantly higher in cirrhosis patients when compared with those of the healthy controls and the pre-cirrhotic groups (*p* < 0.001). Considering only patients with MASLD, the serum levels of GP73 increased in different stages of fibrosis from F0 to F4 (Table 1). Moreover, the AUC indicated that serum GP73 exhibited a potential to differentiate patients at different fibrotic stages in MASLD: the AUC for GP73 to predict significant fibrosis (≥F2), severe fibrosis (≥F3), and cirrhosis (F4) was 0.897, 0.935, and 0.960, respectively.

Zheng et al. [49] evaluated the diagnostic performance of a combined and sequential strategy including GP73 levels and CK18-M30 fragments to predict MASH in MASLD patients with persistently normal ALT levels. The authors reported that serum levels of GP73 were significantly lower in the persistently normal ALT group without MASH and increased in patients with MASH. Furthermore, they showed the ability to discriminate MASH from simple steatosis with serum GP73 levels alone in the subgroup of patients with MASLD with persistently normal ALT, reporting that 58 (55.2%) patients were accurately identified with MASH using serum GP73 alone

### 3.2. Experimental Study

#### 3.2.1. Characteristics of Patients and Serum GP73 Concentration in MASLD Participants with Different Liver Fibrosis Stages

In our experimental study, we determined serum GP73 levels in 84 patients, of which 60 were biopsy-confirmed MASH, and in 15 healthy controls. The main characteristics of the patients are reported in Table 2.

The mean serum GP73 concentrations of MASLD and MASH patients were 30 ± 12 ng/mL and 32 ± 12 ng/mL, respectively, and were significantly higher than that of the control group (19 ± 30 ng/mL) (*p* < 0.05) (Table 3).

For the fibrosis stage, 23 subjects (27.4%) had F0 fibrosis, 20 (23.8%) had F1, 9 (10.7%) had F2, 27 (32.1%) had F3, and 5 (6%) had F4. When considering the fibrosis stage, we considered the following subgroups of patients: patients with no significant fibrosis (F0 + F1, *n* = 42); patients with minor fibrosis (F0 + F1 + F2, *n* = 51); patients with significant fibrosis (F2 + F3 + F4, *n* = 42); and patients with advanced fibrosis (F3 + F4, *n* = 33).

**Figure 3:**
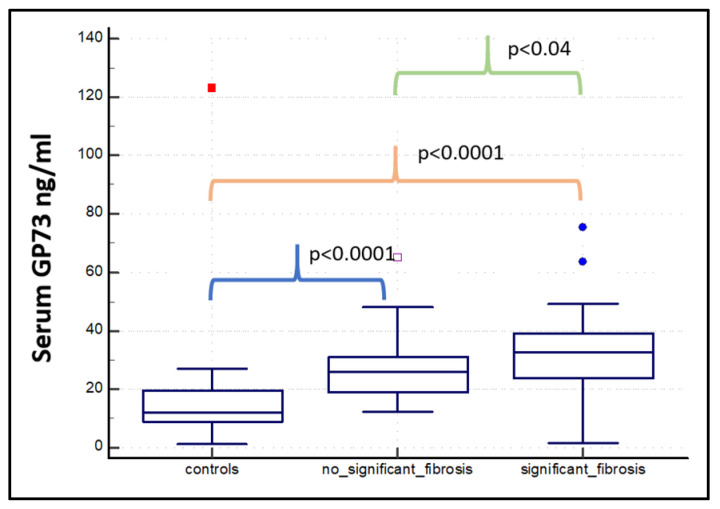
Box plots showing the median serum levels, first and third quartile, and minimum and maximum values of GP73 in the healthy control group (*n* = 15), in patients with no significant fibrosis (F0 + F1, *n* = 42), and in patients with significant fibrosis (F2 + F3 + F4, *n* = 42).

**Figure 4:**
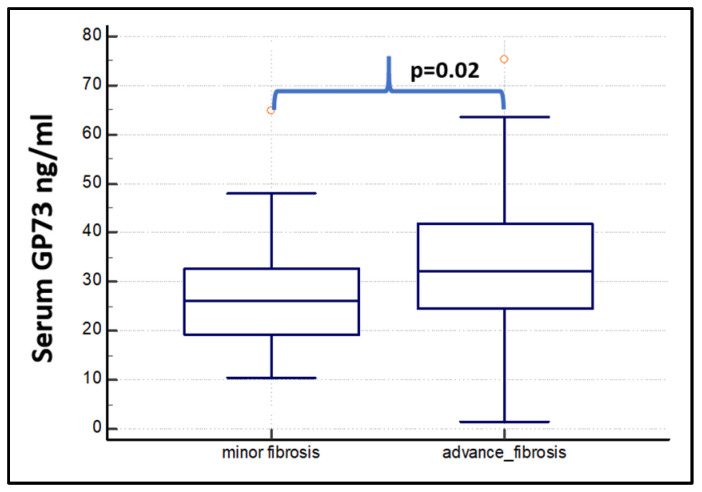
Box plots showing the median serum levels, first and third quartile, and minimum and maximum values of GP73 in patients with minor fibrosis (F0 + F1 + F2, *n* = 51) and in patients with advanced fibrosis (F3 + F4, *n* = 33).

The mean serum GP73 concentration was 25.37 ± 8.9 ng/mL in participants with F0 stage fibrosis, 29.06 ± 11.9 ng/mL for F1 patients, 27.7 ± 10.6 ng/mL for F2, 33.8 ± 14.7 ng/mL for F3, and 32.6 ± 13.6 ng/mL for F4. Considering the individual subgroups, only the F0 vs F3 was statically significant (*p* < 0.05) (Supplementary Table S4). The GP73 serum levels were significantly higher in patients with significant (mean 32 ± 14 ng/mL) and advanced (mean 33 ± 14 ng/mL) fibrosis than in controls and no significant fibrosis (F0 + F1) (Table 3; Figure 3 and Figure 4).

Correlation analysis was performed to determine if elevated serum GP73 levels correlate with other biomarkers of liver disease. The serum GP73 concentrations in participants with NAFLD positively correlated with CIV (r = 0.5; *p* < 0.0001) and PIIIPNP (r = 0.4; *p* < 0.006).

#### 3.2.2. Diagnostic Value of Serum GP73 in Participants with MASLD

**Figure 5:**
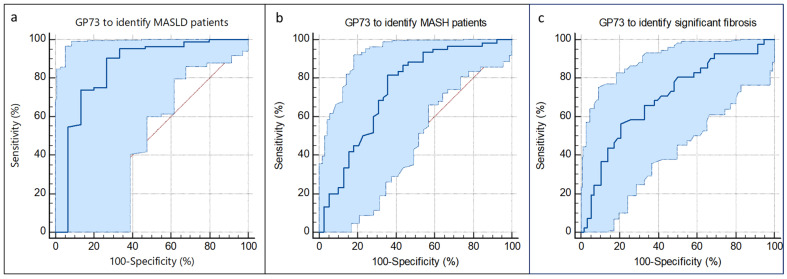
Diagnostic performance of GP73 to identify MASLD (**a**), MASH (**b**), and significant fibrosis (**c**) patients. (**a**) GP73 to identify NAFLD: AUROC (95%CI) 0.85 (0.76–0.91). (**b**) GP73 to identify NASH patients: AUROC (95%CI) 0.75 (0.64–0.82). (**c**) GP73 to identify patients with significant fibrosis: AUROC (95%CI) 0.7 (0.6–0.79). Abbreviations: AUROC, area under receiver operating characteristic; GP73, Golgi protein 73; NASH, non-alcoholic steatohepatitis; MASLD: metabolic dysfunction-associated steatotic liver disease; MASH, metabolic dysfunction-associated steatohepatitis; CI, confidence interval.

The ROC curve suggested that the optimal cut-off value of serum GP73 for identifying patients with MASLD was 15.7 ng/mL, corresponding to an AUROC of 0.85 (95%CI 0.76–0.91), SE of 90.5%, and SP of 73% (Table 4, Figure 5a). Moreover, to identify patients with MASH, significant fibrosis, and advanced fibrosis, the optimal cut-offs were 22.6 ng/mL, 31.1 ng/mL, and 31.1 ng/mL, with corresponding AUROC values of 0.75, 0.7, and 0.67, respectively (Table 4, Figure 5b,c). These data were higher than those of other NITs for all MASH, significant fibrosis, and advanced fibrosis (Table 5).

Likewise, for patients with severe MASH (defined as the presence of MASH, NAS ≥ 4, and fibrosis stage F ≥ 3), the AUROC of GP73 was 0.72 (95% CI 0.6–0.8) with an improved SE at 82% and SP at 62%, much better than those of other NITs (Table 5).

## 4. Discussion

### 4.1. The Role of GP73 as a Biomarker in Diagnosing and Monitoring NAFLD and NASH

Non-alcoholic fatty liver disease (NAFLD) and its progressive form, non-alcoholic steatohepatitis (NASH), present significant global health challenges due to their rising prevalence and potential to progress to cirrhosis and HCC. In 2023, the terms NAFLD and NASH were changed to MASLD and MASH, respectively, to highlight the weight of metabolic and cardiovascular factors in the pathogenesis of these liver disorders [3]. Accurate diagnosis and staging of fibrosis in MASLD/MASH are essential for effective clinical management. While liver biopsy remains the gold standard for diagnosis and staging, its invasive nature, high cost, and susceptibility to sampling errors highlight the need for non-invasive alternatives. Biomarkers, like GP73, have emerged as promising tools for effective and sustainable monitoring of liver disease over the long term in primary care settings where liver biopsy appears impossible to apply in large-scale populations.

In the presence of a liver injury due to hepatitis, autoimmune disease, or viral infection, several chemokines and cytokines are activated, triggering an inflammatory process. Chronic inflammation facilitates the secretion of pro-inflammatory cytokines and interleukins, especially Il-6, aggravating the inflammation and causing an imbalance between fibrogenesis and fibrinolysis in favor of the former and extracellular matrix accumulation [8]. In this context, the secretion of IL-6 enhances the expression of GP73, which, in turn, contributes to the inflammatory pathway activating the HSC and leading to the development of liver fibrosis [9]. The relationship between GP73 and the inflammatory pathway is only partially described. To clarify the molecular mechanism underlying this process, further studies are needed.

In our scoping review, we identified only five studies evaluating GP73 in MASLD patients. All authors showed that the serum concentration of GP73 was higher in patients than in controls, in accordance with the results of our experimental study. Furthermore, the serum GP73 in participants was significantly different (*p* < 0.05) among different severities of hepatic fibrosis [46,48]. In addition, the authors reported good AUROC values of GP73 for predicting significant (0.89) and severe (0.93) fibrosis and cirrhosis (0.96) [48], for the identification of MASH (0.83) patients [46], and to identify severe (0.89) and moderate (0.74) inflammatory activity in MASH patients [47]. Evidence about the diagnostic accuracy of GP73 is still limited. It is necessary to conduct further studies to verify the real accuracy of the biomarker in a well-defined patient population. In terms of the clinical application of markers for MASH or liver fibrosis, GP73 measurements are not widely promoted, probably due to inadequate knowledge about (a) the pathophysiological mechanism underlying the interaction between GP73 and the inflammatory pathway, and (b) the diagnostic accuracy of GP73 in MASLD patients. In our experimental study, we showed a good AUROC value for identifying MASLD patients (0.85), whereas the AUROC values were fair for MASH (0.75) and fibrosis ≥ 2 (0.7), making its use in clinical practice questionable.

Studies evaluating GP73 use for liver fibrosis detection with respect to available biomarkers are scarce and sometimes contradictory. Cao et al. [50] confirmed the performance of GP73 in detecting severe liver fibrosis in patients with chronic HBV (AUC 0.76) and reported better performance than APRI (AUC 0.69) and FIB4 (AUC 0.81). However, Qian et al. [33] reported differing results in HCC patients, reporting that the diagnostic values of GP73 for advanced fibrosis (F ≥ 3, AUC 0.7) and cirrhosis (F = 4, AUC 0.77) were limited when compared to APRI (AUC 0.83 and 0.83, respectively) and FIB-4 (AUC 0.8 and 0.9, respectively). Furthermore, a recent meta-analysis evaluating GP73 in 16 studies enrolling Chinese patients with HCV and HBC confirmed its accuracy as a marker for significant (AUC 0.818), advanced fibrosis (AUC 0.852), and cirrhosis (AUC 0.894) [51]. However, the diagnostic role of GP73 in detecting advanced fibrosis in MASLD patients has not been sufficiently studied, as reported in our scoping review. Liu et al. [10] reported a better accuracy of serum GP73 with respect to APRI for significant liver fibrosis (0.76 vs. 0.67) in children aged below 3 years. Li et al. [46] reported good sensibility (88.9%) and specificity (42.7%), and the ROC curves showed better accuracy for Fibroscan. Yao et al. [48] reported that serum levels of GP73 exhibited better diagnostic value than APRI and FIB-4 in patients with compensated cirrhosis.

### 4.2. Strengths and Weaknesses

In the present study, we aimed to determine the serum concentration of GP73 in MASLD patients and evaluate its diagnostic accuracy and appropriateness as a non-invasive biomarker. We determined the serum GP73 concentration in MASLD patients and healthy controls and showed that the serum GP73 concentration is higher in patients with significant fibrosis with respect to controls and non-significant fibrosis. We also executed a ROC analysis to calculate the AUC to identify patients with MASLD, MASH, or significant fibrosis, finding fair values, and calculated the optimal cut-offs.

The present study had several limitations. First, the sample size was small, and we included only 15 healthy controls, so future studies should recruit a wider number of participants. Second, in our cohort, 42% of the included participants had significant fibrosis (F ≥ 2), 33% had advanced fibrosis, and 5% had cirrhosis; this distribution was divergent from the literature [52]. Likewise, the distribution of patients in fibrosis stage subgroups was different from the estimated prevalence [53], which explains the small difference in GP73 concentrations among the different fibrosis subgroups and could generate a potential selection bias. Third, the biological function of GP73 and the molecular mechanism involved are not yet defined; therefore, additional studies are needed to understand the role of GP73 as a possible biomarker of advanced fibrosis and MASH.

### 4.3. Advantages of GP73 as a Biomarker

GP73 is measurable in serum, offering a non-invasive alternative to liver biopsy, which is often associated with risks such as pain and bleeding. This substantially reduces patient burden while maintaining diagnostic utility. Our literature scoping review on GP73 accuracy indicates that GP73 levels are elevated in patients with liver fibrosis, positioning it as a potential diagnostic marker.

GP73, a glycoprotein upregulated in response to hepatocyte stress and damage, is more specific to liver fibrosis than indirect markers such as the AST/ALT ratio. Studies suggest that GP73 correlates well with fibrosis severity, offering a quantitative measure for disease staging [51]. Furthermore, GP73 has demonstrated promise in detecting fibrosis stages before clinical symptoms manifest, enabling timely interventions to prevent disease progression. Its dynamic monitoring capability allows for repeated measurements to track disease progression or regression over time. Compared to biopsy and advanced imaging techniques, GP73 measurement also provides a cost-effective option, especially in resource-limited settings. Its utility is now possibly relevant due to therapeutic advancements, such as the FDA’s approval of Rezdiffra (Resmetirom) in March 2024, which made treatment options for MASH available for patients with moderate to advanced fibrosis [54,55]. This drug selectively targets hepatic fat accumulation and inflammation, and the ability of GP73 to possibly identify moderate to severe fibrosis may be of value for ensuring appropriate prescription for large-scale populations [56].

Resmetirom (brand name Rezdiffra) received accelerated approval from the U.S. Food and Drug Administration (FDA) in March 2024 for the treatment of adults with MASH with moderate to advanced liver fibrosis to be used along with diet and exercise. Resmetiron is a partial activator of a thyroid hormone receptor, and the activation of this receptor by the drug in the liver reduces liver fat accumulation. To be eligible for this new treatment, patients needed to have a liver biopsy showing inflammation due to MASH with moderate or advanced liver scarring. After 12 months, liver biopsies showed that a greater proportion of subjects who were treated with Resmetiron achieved MASH resolution or an improvement in liver scarring compared with those who received the placebo. As GP73 is a biomarker associated with liver disease progression, it may be proposed for its potential role in monitoring therapeutic responses in MASH patients as a non-invasive and safe diagnostic practice suitable for large populations. To date, the specific role of GP73 compared to all other fibrosis direct biomarkers in monitoring responses to Resmetirom therapy was not highlighted in the approval FDA announcements. However, given GP73’s association with liver fibrosis, this new biomarker is a potential useful candidate for assessing Resmetiron treatment efficacy in evaluating patient responses over time. Further research is warranted to establish standardized protocols for using GP73 in clinical research and clinical practice to monitor treatment outcomes in MASH patients undergoing Resmetirom therapy.

### 4.4. Challenges in GP73 Utilization

Despite its advantages, GP73 is not without limitations. One of the primary challenges is its lack of clinical specificity, as elevated GP73 levels are not exclusive to MASLD or MASH. Similar elevations occur in conditions like viral hepatitis, alcoholic liver disease, and HCC [57]. This necessitates complementary diagnostic tools to rule out alternative causes.

In our experimental study, a concentration ≥ 45 ng/mL was indicated by manufacturers as a positive result, but our statistical analysis identified optimal cut-offs of 15.7 and 22.6 to identify MASLD and MASH patients. These data suggest a revision of the indications of the manufacturers to optimize the clinical use of the test.

Standardization issues further complicate GP73 clinical application. There is no universally accepted cut-off value, leading to variability in diagnostic accuracy across studies and populations [39]. Assay differences and a lack of harmonized measurement protocols limit its reproducibility and consistency. A more pragmatic approach, as suggested in our research, involves defining context-specific cut-off values based on clinical objectives, such as early detection of fibrosis when requested or evaluation of progression to advanced stages as in the case of previously reported new therapeutic options. Establishing decisional thresholds, rather than a “normal reference value”, may enhance GP73 integration into clinical workflows promoting its effectiveness.

Variability in GP73 expression among different ethnicities and populations also affects its sensitivity and specificity [39]. Moreover, common comorbid conditions in MASLD/MASH patients, such as diabetes, obesity, and metabolic syndrome, influence GP73 levels and complicate interpretation [58]. Lastly, while cross-sectional studies support GP73’s role in fibrosis staging, longitudinal data on its predictive value for disease progression or therapeutic response remain limited.

The use of GP73 to evaluate the progression of liver fibrosis could add value in patient management. Current evidence about the clinical use of GP73 is still scarce. Although some studies showed the involvement of GP73 in liver disease, few of them reported information about the accuracy of GP73 to differentiate advanced from no or minimal fibrosis. Szternel et al. [7] suggested that GP73 should be applied in patients at risk of developing liver disease as a first-level test for ruling out advanced fibrosis or as a second-level test for confirming advanced fibrosis. The strategies combining GP73 with other biomarkers could improve its diagnostic accuracy in identifying advanced fibrosis and provide precise information about liver fibrosis stage, but further studies are needed to establish its position in an algorithm (triage, add-on, or replacement) and to confirm the clinical relevance of this biomarker.

## 5. Conclusions

GP73 is a promising direct fibrosis biomarker with potential applications in diagnosing and staging liver fibrosis in MASLD and MASH, as supported by our scoping review and our experimental research. Its non-invasive nature, correlation with fibrosis severity, and potential role in long-term monitoring make it an attractive candidate for clinical use. However, issues such as lack of standardization, limited specificity, and variability across populations must be addressed before its widespread implementation in clinical practice.

Future research should prioritize the standardization of assay protocols and measurement techniques, the validation of GP73 across diverse populations, and the definition of its role in diagnostic algorithms alongside other biomarkers and imaging techniques to explore its utility in monitoring disease progression and evaluating therapeutic efficacy, particularly in the context of emerging treatments like Resmetirom.

Through these efforts, GP73 could become a tool for non-invasive liver fibrosis assessment, aiding in the comprehensive management of MASLD and MASH.

## Figures and Tables

**Table 1:** Main characteristics of studies included in the scoping review.

Authors, Year	Objective	Patients	Method to Detect Serum GP73	Serum GP73, ng/mL	Cut-Off Value for GP73	AUROC	Sensitivity	Specificity
Li, 2021 [46]	To determinate the concentrations of GP73 in the serum and liver of patients with MASLD	91 patients with MASLD (46 NAFL and 45 MASH) and 30healthy controls	ELISA	NAFL: 47.92 ± 9.83 MASH: 62.17 ± 10.79 CTRs: 43.29 ± 5.9	Fibrosis ≥ 2: 50.2 ng/ml	Diagnosis of MASH: 0.83	Fibrosis ≥ 2: 88.9%	Fibrosis ≥ 2: 42.7%
Liu, 2018 [10]	To explore the value of serum GP73 levels in diagnosing significant fibrosis in children	age < 3 y *n* = 116 S0–S1 *n* = 31 S2–S4 *n* = 85	ELISA	age < 3 y:218.7 ± 88.0 CTRs < 3 y: 172.9± 84.2 S0–S1: 162 ± 78.5 S2–S4: 239 ± 82.3	age < 3 y: 179.6 ng/mL	age < 3 y: 0.76 (95% CI: 0.66–0.86)	age < 3 y: 76.5%	age < 3 y: 67.7%
		age ≥ 3 y *n* = 67 S0–S1 *n* = 26 S2–S4 *n*= 41		age ≥ 3 y: 141.4 ± 68.8 CTRs ≥ 3 y 56.3 ± 23.4 S0–S1: 128.4 ± 81.7 S2–S4: 149.6 ± 58.8	age ≥ 3 y: 102.9 ng/mL	age ≥ 3 y: 0.62 (95%CI: 0.47–0.77)	age ≥ 3 y: 82.9%	age ≥ 3 y: 46.2%
Wang, 2020 [47]	1. To determine the correlation between serum GP73 and the severity of hepatic necroinflammation 2. To evaluate the diagnostic performance of serum GP73	MASH: 201 (125 as mild inflammatory activity (≤G1), 67 as moderate activity (G = 2), 9 (4.5%) as severe activity (G = 3))	ELISA	G0–G1: median 49.98 (31.49–75.05) ng/mL; G2: median 76.61 (48.68–110.03) G3: median 116.44 (103.41–162.17) ng/mL	For G ≥ 2 94.57 ng/mL For G = 3: 101.1 ng/mL	For G ≥ 2: 0.742 95% CI: 0.676–0.801 For G = 3: 0.891 95%CI: 0.840–0.931	For G ≥ 2: 44.74% For G = 3: 88.89%	For G ≥ 2: 92.80% For G = 3: 85.94%
Yao, 2018 [48]	To examine the potential value of serum GP73 in diagnosing chronic liver disease both in cirrhotic and pre-cirrhotic subjects	3044 (pre-cirrhosis *n* = 956, compensated cirrhosis *n* = 1247, decompensated cirrhosis *n* = 841) 121 healthy volunteers as controls MASLD *n* = 143	ELISA	Pre-cirrhotic: median 43.74 (IQR 28.24–61.34) ng/mL Compensated cirrhotic: median 122.00 (IQR 82.33–181.28) ng/mL Decompensated cirrhotic: median 149.85 (IQR 95.49–215.70) ng/mL CTRs: median 35.07 (IQR 24.97–45.37) ng/mL MASLD PATIENTS FO-F1: 32.54 (22.91–48.68) ng/mL, F2: 54.85 (45.72–77.12) ng/mL, F3: 69.52 (53.19–94.88) ng/mL, F4 127.60 (98.43–211.40) ng/mL	MASLD F ≥ 2: 59.17 F ≥ 3: 59.17 F4: 77.12	Compensated cirrhosis: 0.899 95%CI 0.886–0.911 Cirrhosis in MASLD: 0.962 95%CI 0.917–0.987 MASLD F ≥ 2: 0.897 (95%CI 0.835–0.941) F ≥ 3: 0.935 (95%CI 0.881–0.969) F4: 0.96 (95%CI 0.914–0.986)	MASLDF ≥ 2: 76.27% F ≥ 3: 92.5% F4: 89.29%	MASLD F ≥ 2: 88.10% F ≥ 3: 82.52% F4: 90.43
Zheng, 2020 [49]	To test the diagnostic performance of GP73 in predicting NASH in patients with normal ALT (nALT) levels.	MASLD: 345 (MASH: 240) nALT: 105 (MASH: 53), abnALT: 240 (MASH: 187)	ELISA	Overall: 80.96 ± 40.05 abnALT: 85.55 ± 43.02 nALT: 70.45 ± 29.88 MASH: 76.20 ± 35.21 noMASH: 64.59 ± 22.07	53 ng/mL	Accuracy: 55.2%	77.3%	32.7–5

Data are reported as mean ± standard deviation. Abbreviations: MASLD, metabolic dysfunction-associated steatotic liver disease; MASH, metabolic dysfunction-associated steatohepatitis; NAFL, non-alcoholic fatty liver disease; CTRs, controls; S0–S1, no or minor fibrosis; S2–S4, significant fibrosis; CI, confidence interval; IQR, interquartile range; y, years; ALT, alanine transaminase; nALT, normal ALT; abnALT, above the upper limit of normal ALT; ELISA, enzyme-linked immunosorbent assay.

**Table 2:** Characteristics of patients with MASLD included in our experimental study.

	MASLD
*N*	84
Gender (M/F)	60/24
Age (years)	51 ± 11.5
ALT (U/L)	56 ± 38
AST (U/L)	40 ± 22
GGT (U/L)	78 ± 119
PLT (×10^9^/L)	214.8 ± 57.8
AST/ALT	0.8 ± 0.4
APRI	0.6 ± 0.4

Data are reported as mean ± standard deviation. Abbreviations: MASLD, metabolic dysfunction-associated steatotic liver disease; ALT, alanine aminotransferase activity; AST, aspartate aminotransferase activity; GGT, gamma glutamyl transferase activity; PLT, platelet count.; APRI, AST to Platelet Ratio Index; U/L, units/liter.

**Table 3:** Serum concentration of GP73 (ng/mL) in different cohorts of patients.

	*N*	Mean (ng/mL)	SD
All patients	84	29.64	12.38
NASH patients	60	31.52	12.78
Healthy controls	15	19.23	29.67
Patients with significant fibrosis (F2 + F3 + F4)	42	32.36	13.53
Patients with advanced fibrosis (F3 + F4)	33	33.33	14.23
Patients with no significant fibrosis (F0 + F1)	42	26.9	10.58
Patients with minor fibrosis (F0 + F1 + F2)	51	27.25	10.48
Patients with F0	23	25.37	8.9
Patients with F1	20	29.06	11.9
Patients with F2	9	27.7	10.6
Patients with F3	27	33.8	14.7
Patients with F4	5	32.6	13.6

Data are reported as the mean expressed in ng/mL and standard deviation (SD). Abbreviations: F0, fibrosis stage 0; F1, fibrosis stage 1; F2, fibrosis stage 2; F3, fibrosis stage 3; F4, fibrosis stage 4.

**Table 4:** Diagnostic value of GP73 in patients.

	Cut-Off Value * (ng/mL)	AUROC (95%CI)	Sensitivity (95%CI)	Specificity (95%CI)	Positive Likelihood Ratio (95%CI)	Negative Likelihood Ratio (95%CI)
MASLD	15.7	0.85 (0.76–0.91)	90.5% (82.1–95.8)	73.3% (45–92.2)	3.4 (1.5–7.9)	0.13 (0.06–0.27)
MASH	22.6	0.75 (0.64–0.82)	81.7% (69.6–90.5)	64.1 (42.2–78.8)	2.28 (1.47–3.52)	0.29 (0.16–0.51)
Significant fibrosis (F2 + F3 + F4)	31.1	0.7 (0.6–0.79)	56.1% (39.7–71.5)	79.3% (66.6–88.8)	2.71 (1.53–4.8)	0.55 (0.38–0.8)
Advanced fibrosis (F3 + F4)	31.1	0.67 (0.56–0.77)	59.4% (40.6–76.3)	71.2% (57–83)	2.06 (1.23–3.4)	0.57 (0.36–0.9)
Severe MASH	26.1	0.72 (0.62–0.8)	82.1% (63.1–93.9)	62% (49.7–73.2)	2.16 (1.53–3.05)	0.29 (0.13–0.65)

* The cut-off values were calculated using Youden’s index. Abbreviations: MASLD, metabolic dysfunction-associated steatotic liver disease; MASH, metabolic dysfunction-associated steatohepatitis; AUROC, area under receiver operating characteristic curve.

**Table 5:** AUROC and 95%CI values of simple and complex NITs in subgroup patients.

	MASH	Significant Fibrosis (F2 + F3 + F4)	Advanced Fibrosis (F3 + F4)	Severe MASH
*N*	60	41	32	28
AST	0.58 (0.47–0.69)	0.59 (0.47–0.69)	0.61 (0.5–0.7)	0.6 (0.5–0.7)
ALT	0.55 (0.44–0.66)	0.5 (0.4–0.6)	0.5 (0.4–0.62)	0.54 (0.43–0.66)
GGT	0.52 (0.41–0.63)	0.53 (0.46–0.65)	0.5 (0.4–0.63)	0.57 (0.46–0.68)
PLT	0.53 (0.41–0.64)	0.56 (0.45–0.67)	0.6 (0.47–0.69	0.51(0.4–0.6)
AST/ALT	0.52 (0.41–0.63)	0.6 (0.5–0.7)	0.67 (0.56–0.77)	0.55 (0.44–0.66)
APRI	0.56 (0.45–0.67)	0.6 (0.5–0.7)	0.64 (0.53–0.74)	0.6 (0.5–0.7)

Abbreviations: *N*, number; MASH, metabolic dysfunction-associated steatohepatitis; ALT, alanine aminotransferase activity; AST, aspartate aminotransferase activity; GGT, gamma glutamyl transferase activity; PLT, platelet count.; APRI, AST to platelet ratio index.

## Data Availability

The raw data supporting the conclusions of this article will be made available by the authors on request.

## Supplementary Materials

The following supporting information can be downloaded at: https://www.mdpi.com/article/10.3390/diagnostics15050544/s1. Table S1: Details of literature search strategies; Table S2: References for excluded articles, with reasons; Table S3: Methodological quality assessment of included studies using QUADAS-2; Table S4: *p*-values of comparisons among fibrosis subgroups calculated using the Mann–Whitney test.

## Abbreviations

The following abbreviations are used in this manuscript:

ALT	Alanine aminotransferase
APRI	AST to platelet ratio index
AST	Aspartate aminotransferase
AUROC	Area under the receiver operating characteristic
CIV	Collagen type IV
GGT	Gamma glutamyl transferase
GP73	Golgi protein 73
HA	Hyaluronic acid
HCC	Hepatocellular carcinoma
LR+	Likelihood ratio positive
LR−	Likelihood ratio negative
MASLD	Metabolic dysfunction-associated steatotic liver disease
MASH	Metabolic dysfunction-associated steatohepatitis
NAFLD	Non-alcoholic fatty liver disease
NASH	Non-alcoholic steatohepatitis
NITs	Non-invasive test
PIIIPNP	Amino terminal peptide of type III procollagen
PLT	Platelet count
ROC	Receiver operating characteristic
SE	Sensitivity
SP	Specificity
