# Supplementary material for: Diagnostic Accuracy of Golgi Protein 73 (GP73) for Liver Fibrosis Staging in Metabolic Dysfunction-Associated Steatotic Liver Disease: A Scoping Review and Cohort Study
Source: Diagnostics (Basel). 2025 Feb 24;15(5):544. doi: 10.3390/diagnostics15050544 (PMC11898419; doi:10.3390/diagnostics15050544)
Supplement: Supplementary file 1 [file diagnostics-15-00544-s001.zip › diagnostics-3431487-supplementary.pdf]

Supplemental Tables

Table S1: Detail of literature search strategies

EMBASE – novembre 2024	((TITLE-ABS-KEY ("Liver Cirrhosis")) OR (TITLE-ABS-KEY (fibrosis)) OR (TITLE-ABS-KEY ("Metabolic dysfunction-associated fatty liver disease")) OR (TITLE-ABS-KEY ("Non-alcoholic Fatty Liver disease"))) AND (TITLE-ABS-KEY ("Golgi protein 73" OR gp73))
PUBMed – novembre 2024	((Golgi protein 73 OR GP73)) AND ((((((("Non-alcoholic Fatty Liver Disease/diagnosis"[Mesh] OR "Non-alcoholic Fatty Liver Disease/pathology"[Mesh] OR "Non-alcoholic Fatty Liver Disease/physiopathology"[Mesh])) OR ("Non-alcoholic Fatty Liver Disease" OR NAFLD)) OR ("Metabolic dysfunction-associated fatty liver disease" OR nafld)) OR ("Non-alcoholic Fatty Liver Disease" OR NAFLD)) OR ("Metabolic dysfunction-associated fatty liver disease" OR nafld)) OR (("Non-alcoholic Fatty Liver Disease/diagnosis"[Mesh] OR "Non-alcoholic Fatty Liver Disease/pathology"[Mesh] OR "Non-alcoholic Fatty Liver Disease/physiopathology"[Mesh]))) OR ((((("Fibrosis/blood"[Mesh] OR "Fibrosis/diagnosis"[Mesh] OR "Fibrosis/pathology"[Mesh] OR "Fibrosis/physiopathology"[Mesh])) OR (("Liver Cirrhosis/blood"[Mesh] OR "Liver Cirrhosis/diagnosis"[Mesh] OR "Liver Cirrhosis/pathology"[Mesh] OR "Liver Cirrhosis/physiopathology"[Mesh]))) OR ("liver fibrosis"))))

Table S2: Reference of excluded articles with reason

Reference	Reason of exclusion
Atilla, A.; Taşkın, M.H.; Kazak, Z.; Aydın, S.; Kılıç, S.S. GP73 level in patients with chronic hepatitis B: Relationship with liver biopsy, levels of ALT, AST and HBV DNA. Indian J Pathol Microbiol. 2022, 65:55-58. doi: 10.4103/IJPM.IJPM_1149_20. [18]	No population of interest
Bröker, M.E.; Ijzermans, J.N.; Witjes, C.D.; van Vuuren, H.J.; de Man, R.A. The predictive value of Golgi protein 73 in differentiating benign from malignant liver tumors. PLoS One. 2014, 9, e100187. doi: 10.1371/journal.pone.0100187. [19]	No population of interest
Gatselis, N.K.; Tornai, T.; Shums, Z.; Zachou, K.; Saitis, A.; Gabeta, S.; Albesa, R.; Norman, G.L.; Papp, M.; Dalekos, G.N. Golgi protein-73: A biomarker for assessing cirrhosis and prognosis of liver disease patients. World J Gastroenterol. 2020, 26, :5130-5145. doi: 10.3748/wjg.v26.i34.5130. [20]	No population of interest
Gatselis, N.K.; Zachou, K.; Giannoulis, G.; Gabeta, S.; Norman, G.L.; Dalekos, G.N. Serum Cartilage Oligomeric Matrix Protein and Golgi Protein-73: New Diagnostic and Predictive Tools for Liver Fibrosis and Hepatocellular Cancer? Cancers (Basel). 2021, 13, 3510. doi: 10.3390/cancers13143510. [21]	No population of interest
Hemken, P.M.; Qin, X.; Sokoll, L.J.; Jackson, L.; Feng, F.; Li, P.; Gawel, S.H.; Tu, B.; Lin, Z.; Hartnett, J. et al., Validation of the novel GLAS algorithm as an aid in the detection of liver fibrosis and cirrhosis based on GP73, LG2m, age, and sex. Clin Proteomics. 2023, 20, 53. doi: 10.1186/s12014-023-09444-7. [22]	No study design
Hui-Ling, C.; Kang-Ming, H.; Yu, Z.; Yin-Han, D.; Huang, D.; Shu-Ping, X.; Hong-Bin C. The potential value of serum GP73 in the ancillary diagnosis and grading of liver cirrhosis. Scand J Clin Lab Invest. 2023, 83, 95-102. doi: 10.1080/00365513.2023.2175238. [23]	No population of interest
Iftikhar, R.; Kladney, R.D.; Havlioglu, N.; Schmitt-Gräff, A.; Gusmirovic, I.; Solomon, H.; Luxon, B.A.; Bacon, B.R.; Fimmel, C.J. Disease- and cell-specific expression of GP73 in human liver disease. Am J Gastroenterol. 2004, 99, 1087-95. doi: 10.1111/j.1572-0241.2004.30572.x. [24]	No population of interest
Jiao. C.; Cui, L.; Piao, J.; Qi, Y.; Yu, Z. Clinical significance and expression of serum Golgi protein 73 in primary hepatocellular carcinoma. J Cancer Res Ther. 2018, 14, 1239-1244. doi: 10.4103/0973-1482.199784. [25]	No population of interest
Jing, J.S.; Ye, W.; Jiang, Y.K.; Ma, J.; Zhu, M.Q.; Ma, J.M.; Zhou, H.; Yu, L.Q.; Yang, Y.F.; Wang, S.C. The Value of GPC3	No population of interest

and GP73 in Clinical Diagnosis of Hepatocellular Carcinoma. Clin Lab. 2017, 63, 1903-1909. doi: 10.7754/Clin.Lab.2017.170712. [26]	
Kohlhepp, M.S.; Liu, H.; Tacke, F.; Guillot, A. The contradictory roles of macrophages in non-alcoholic fatty liver disease and primary liver cancer-Challenges and opportunities. Front Mol Biosci. 2023, 10, 1129831. doi: 10.3389/fmolb.2023.1129831. [27]	No outcome of interest
Liu, M.Y.; Huang, L.; Wu, J.F.; Zhang, H.B.; Ai, W.B.; Zhang, R.T. Possible roles of Golgi protein-73 in liver diseases. Ann Hepatol. 2022, 27, 100720. doi: 10.1016/j.aohep.2022.100720 [28]	Review
Liu, S.; Ma, J.; Chen, P.; Liu, S.; Guo, Y.; Tan, M.; Guo, X.; Feng, Y.; Wang, Q.; Li, W.; et al. Novel serum biomarker of Golgi protein 73 for the diagnosis of clinically significant portal hypertension in patients with compensated cirrhosis. J Med Virol. 2024, 96, e29380. doi: 10.1002/jmv.29380. [29]	No population of interest
Mamdouh, S.; Soliman, A.; Khorshed, F.; Saber, M. Glypican-3, Vascular Endothelial Growth Factor and Golgi Protein-73 for Differentiation between Liver Cirrhosis and Hepatocellular Carcinoma. Asian Pac J Cancer Prev. 2023, 24, 497-507. doi: 10.31557/APJCP.2023.24.2.497. [30]	No population of interest
Mao, Y.; Yang, H.; Xu, H.; Lu, X.; Sang, X.; Du, S.; Zhao, H.; Chen, W.; Xu, Y.; Chi, T.; et al. Golgi protein 73 (GOLPH2) is a valuable serum marker for hepatocellular carcinoma. Gut. 2010, 59, 1687-93. doi: 10.1136/gut.2010.214916. [31]	No population of interest
Marrero, J.A.; Romano, P.R.; Nikolaeva, O.; Steel, L.; Mehta, A.; Fimmel, C.J.; Comunale, M,A.; D'Amelio, A.; Lok, A.S.; Block, T.M. GP73, a resident Golgi glycoprotein, is a novel serum marker for hepatocellular carcinoma. J Hepatol. 2005, 43, 1007-12. doi: 10.1016/j.jhep.2005.05.028 [32]	No population of interest
Peng, Y.; Zeng, Q.; Wan, L.; Ma, E.; Li, H.; Yang, X.; Zhang, Y.; Huang, L.; Lin, H.; Feng, J.; et al. GP73 is a TBC-domain Rab GTPase-activating protein contributing to the pathogenesis of non-alcoholic fatty liver disease without obesity. Nat Commun. 2021, 12, 7004. doi: 10.1038/s41467-021-27309-1. [33]	No study design
Qian, X.; Zheng, S.; Wang, L.; Yao, M.; Guan, G.; Wen, X.; Zhang, L.; Xu, Q.; Chen, X.; Zhao, J.; et al. Exploring the Diagnostic Potential of Serum Golgi Protein 73 for Hepatic Necroinflammation and Fibrosis in Chronic HCV Infection with Different Stages of Liver Injuries. Dis Markers. 2019, 2019, 3862024. doi: 10.1155/2019/3862024. [34]	No population of interest
Qiao, Y.; Chen, J.; Li, X.; Wei, H.; Xiao, F.; Chang, L.; Zhang, R.; Hao, X.; Wei, H. Serum gp73 is also a biomarker for diagnosing cirrhosis in population with chronic HBV infection. Clin Biochem. 2014, 47, 216-22. doi: 10.1016/j.clinbiochem.2014.08.010. [35]	No population of interest
Szternel, Ł.; Sobucki, B.; Wieprzycka, L.; Krintus, M.; Panteghini M. Golgi protein 73 in liver fibrosis. Clin Chim Acta. 2025, 565, 119999. doi: 10.1016/j.cca.2024.119999. [36]	Review
Tian. L.; Wang, Y.; Xu, D.; Gui, J.; Jia, X.; Tong, H.; Wen, X.; Dong, Z.; Tian, Y. Serological AFP/Golgi protein 73 could be a new diagnostic parameter of hepatic diseases. Int J Cancer. 2011, 129, 1923-31. doi: 10.1002/ijc.25838. [37]	No population of interest
Wang, Y.; Wan, Y.Y. Golgi protein 73, hepatocellular carcinoma and other types of cancers. Liver Res. 2020, 4, 161-167. doi: 10.1016/j.livres.2020.09.003 [38]	Review
Wei, H.; Li, B.; Zhang, R.; Hao, X.; Huang, Y.; Qiao, Y.; Hou, J.; Li, X.; Li, X. Serum GP73, a marker for evaluating progression in patients with chronic HBV infections. PLoS One. 2013, 8, e53862. doi: 10.1371/journal.pone.0053862 [39]	No population of interest
Wei, M.; Xu, Z.; Pan, X.; Zhang, X.; Liu, L.; Yang, B.; Chen, Y. Serum GP73 - An Additional Biochemical Marker for Liver Inflammation in Chronic HBV Infected Patients with Normal or Slightly Raised ALT. Sci Rep. 2019, 9,1170. doi: 10.1038/s41598-018-36480-3. [40]	No population of interest
Xia, Y.; Zhang, Y.; Shen, M.; Xu, H.; Li, Z.; He, N. Golgi protein 73 and its diagnostic value in liver diseases. Cell Prolif. 2019, 52, e12538. doi: 10.1111/cpr.12538. [41]	Review
Xiao, J.; Long, F.; Peng, T.; Hu, L.B.; Cai, H.; Chen, R.; Chen, W.L. Development and potential application of a simultaneous multiplex assay of Golgi protein 73 and alpha-fetoprotein for hepatocellular carcinoma diagnosis. Eur Rev Med Pharmacol Sci. 2019, 23, 3302-3310. doi: 10.26355/eurrev_201904_17692. [42]	No population of interest
Xu, Z.; Pan, X.; Wei, K.; Ding, H.; Wei, M.; Yang, H.; Liu, Q. Serum Golgi protein 73 levels and liver pathological grading in cases of chronic hepatitis B. Mol Med Rep. 2015, 11, 2644-52. doi: 10.3892/mmr.2014.3114. [43]	No population of interest
Xu, Z.; Shen, J.; Pan, X.; Wei, M.; Liu, L.; Wei, K.; Liu, L.; Yang, H.; Huang, J. Predictive value of serum Golgi protein 73 for prominent hepatic necroinflammation in chronic HBV infection. J Med Virol. 2018, 90, 1053-1062. doi: 10.1002/jmv.25045. [44]	No population of interest
Yao, M.J.; Wang, L.J.; Liu, S.; Lu, F.M. Application of serum Golgi protein-73 in the management of chronic liver disease. Chin Med J (Engl). 2020, 134, 777-779. doi: 10.1097/CM9.0000000000001296. [45]	No population of interest
Yao, M.; Wang, L.; Wang, J.; Liu, Y.; Liu, S.; Zhao, J.; Lu, F. Diagnostic Value of Serum Golgi Protein 73 for Liver Inflammation in Patients with Autoimmune Hepatitis and Primary Biliary Cholangitis. Dis Markers. 2022, 2022, 4253566. doi: 10.1155/2022/4253566. [46]	No population of interest
Zhang, X.; Wu, L.N.; Li, X.Q.; Luo, X.; Liu, S.W.; Zhang, L.; Nawaz, S.; Ma, L.N.; Ding, X.C. Whether the Golgi protein 73 could be a diagnostic serological marker in hepatocellular carcinoma: a meta analysis. BMC Gastroenterol. 2023, 23, 85. doi: 10.1186/s12876-023-02685-8. [47]	Systematic review

Table S3: Methodological quality assessment of included studies using QUADAS-2

Study	**Patients selection**		**Index test**		**Reference Standard**		**Flow and Timing**
	**Rish of Bias**	**Applicability**	**Rish of Bias**	**Applicability**	**Rish of Bias**	**Applicability**	**Risk of bias**
Li 2021	unclear	low	unclear	low	unclear	low	low
Liu 2018	low	low	low	low	low	low	low
Wang 2020	low	low	low	low	low	low	low
Yao 2018	unclear	low	low	low	low	low	low
Zheng 2020	low	low	low	low	low	low	low

Table S4: p-values of comparison among fibrosis subgroups calculated with the Mann-Whitney test

	F0, n=23	F1, n=20	F2, n=9	F3, n=27
F1	0.27			
F2	0.47	0.98		
F3	**0.0099**	0.13	0.3	
F4	0.15	0.27	0.36	0.85

In bold is reported the statistically significant comparison
